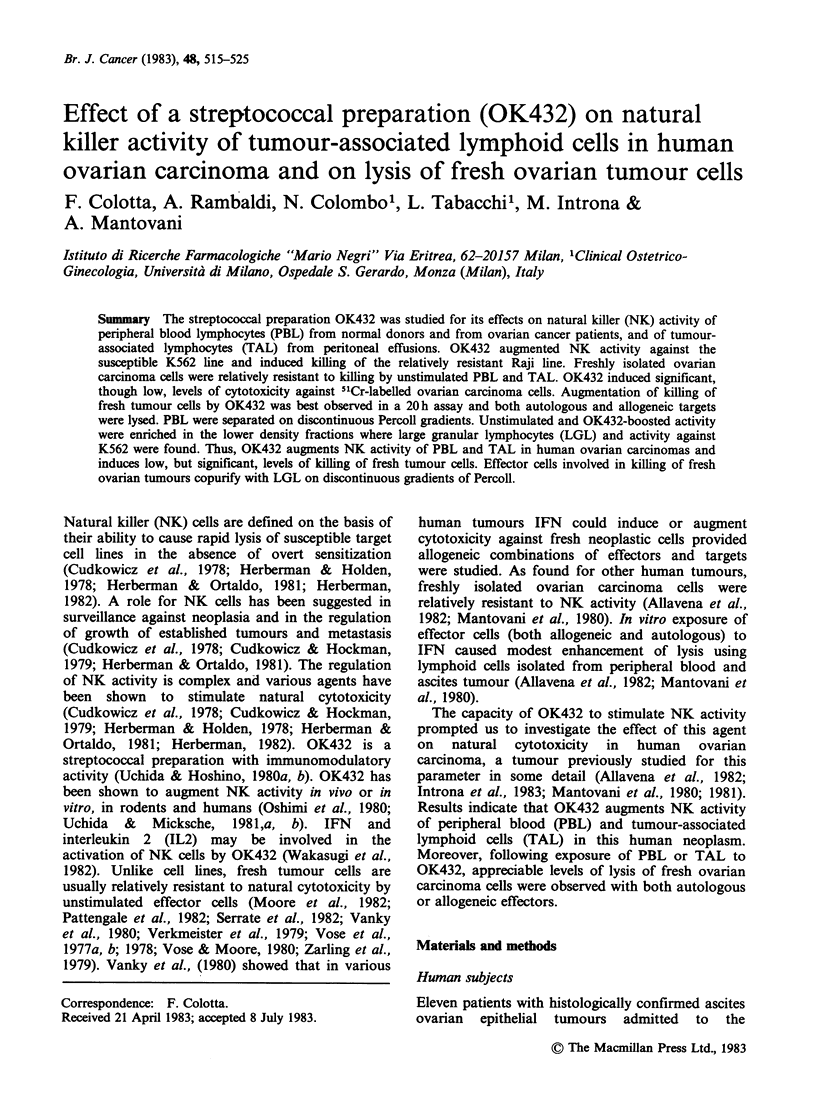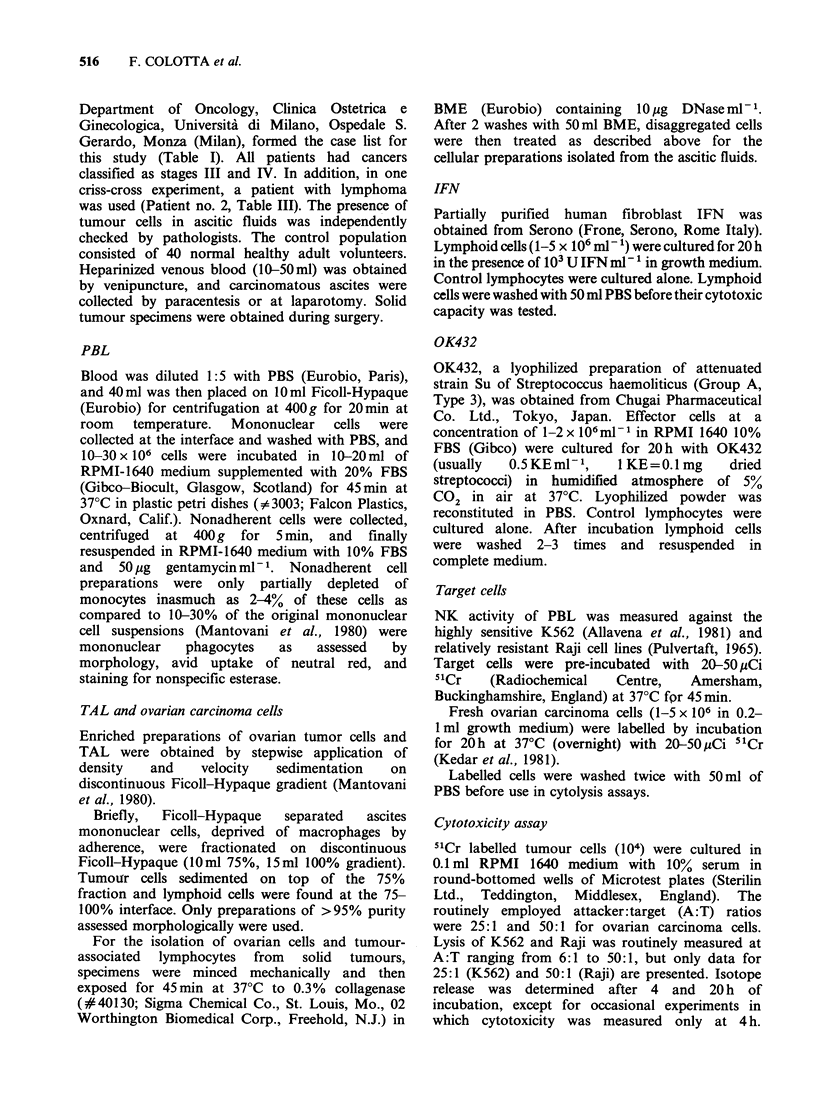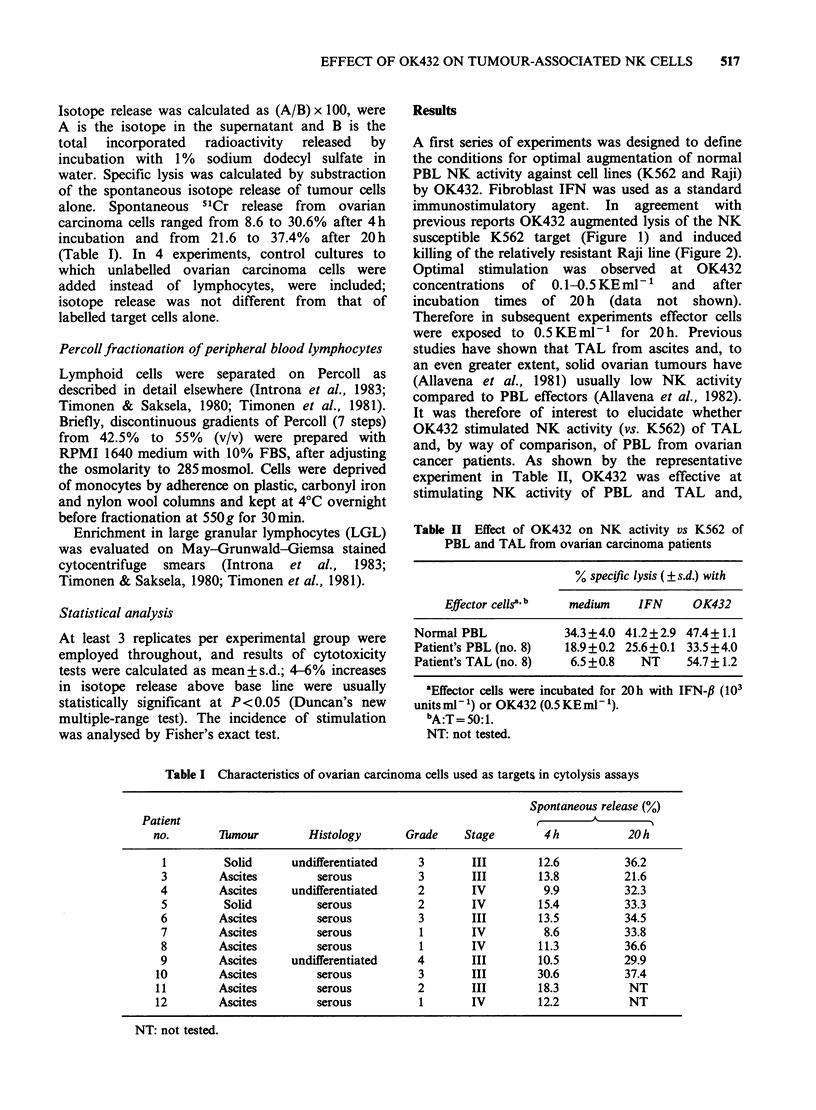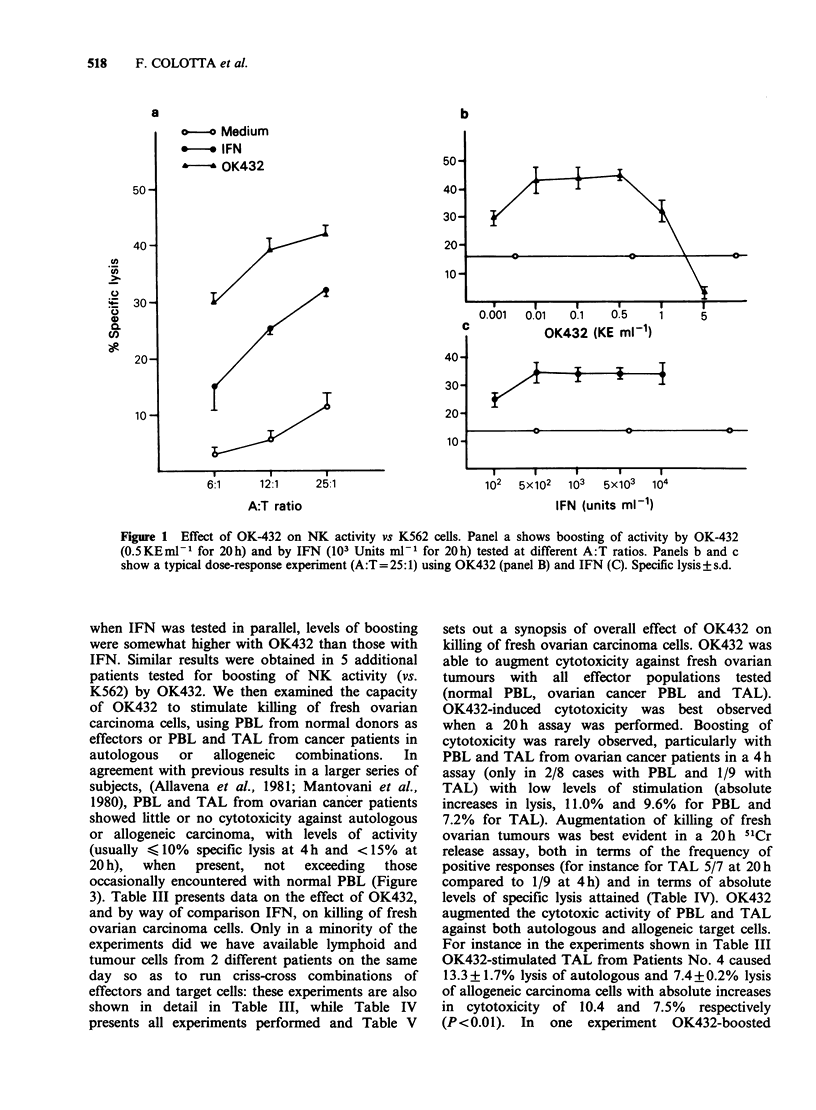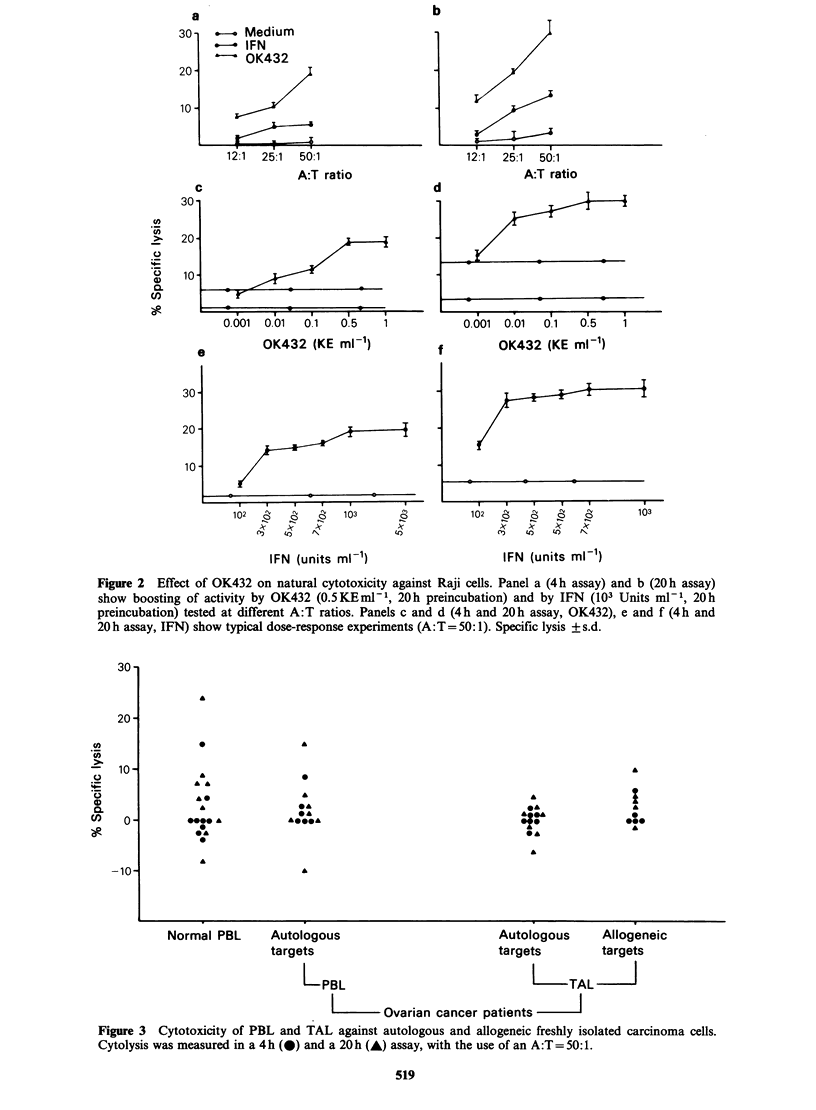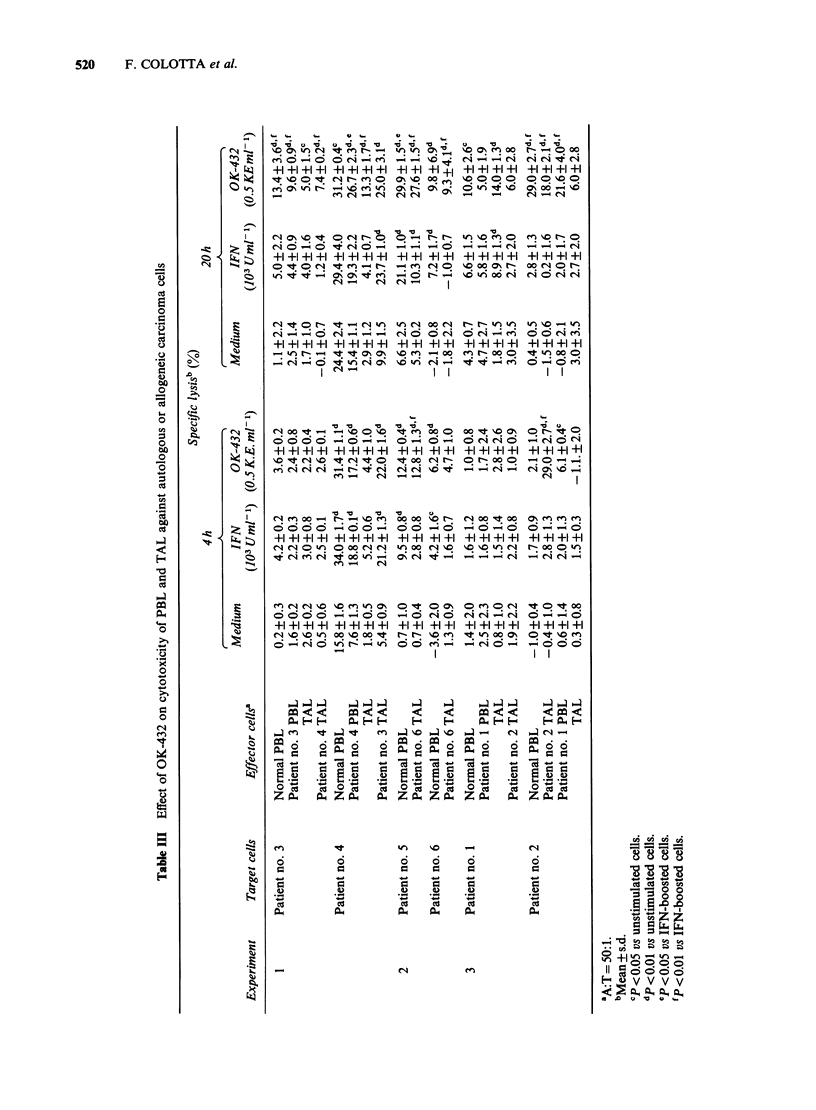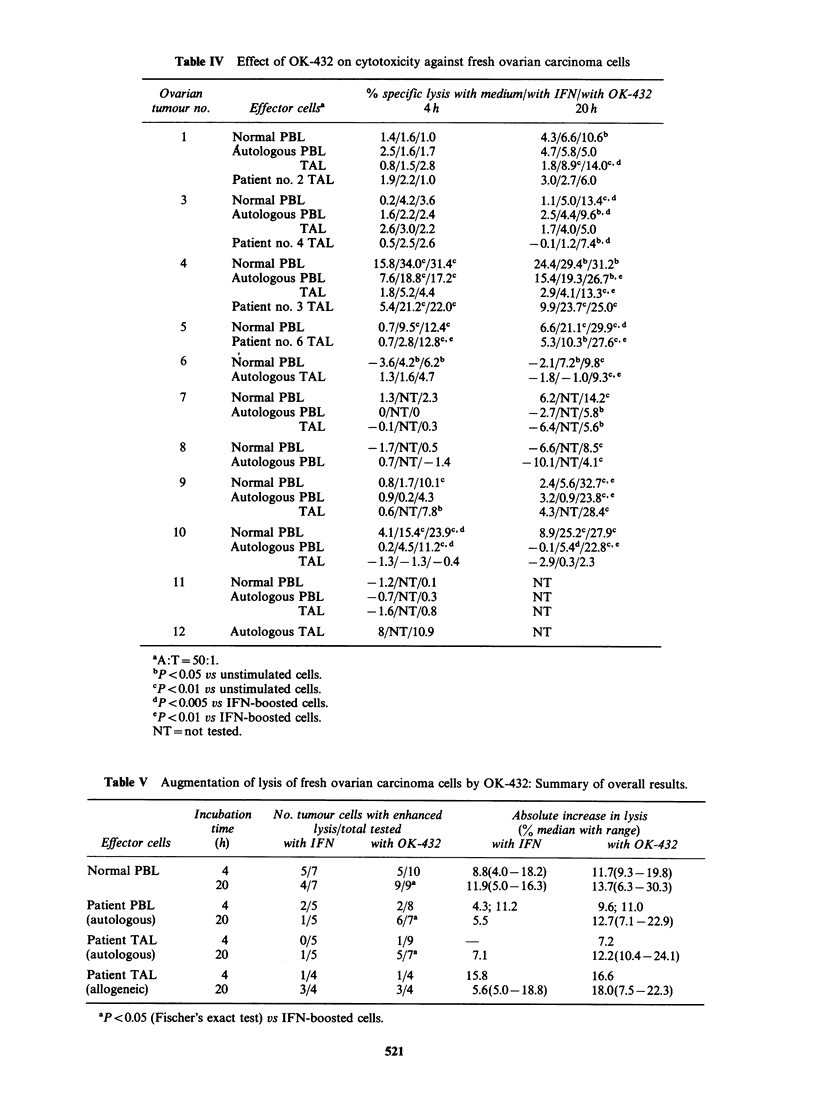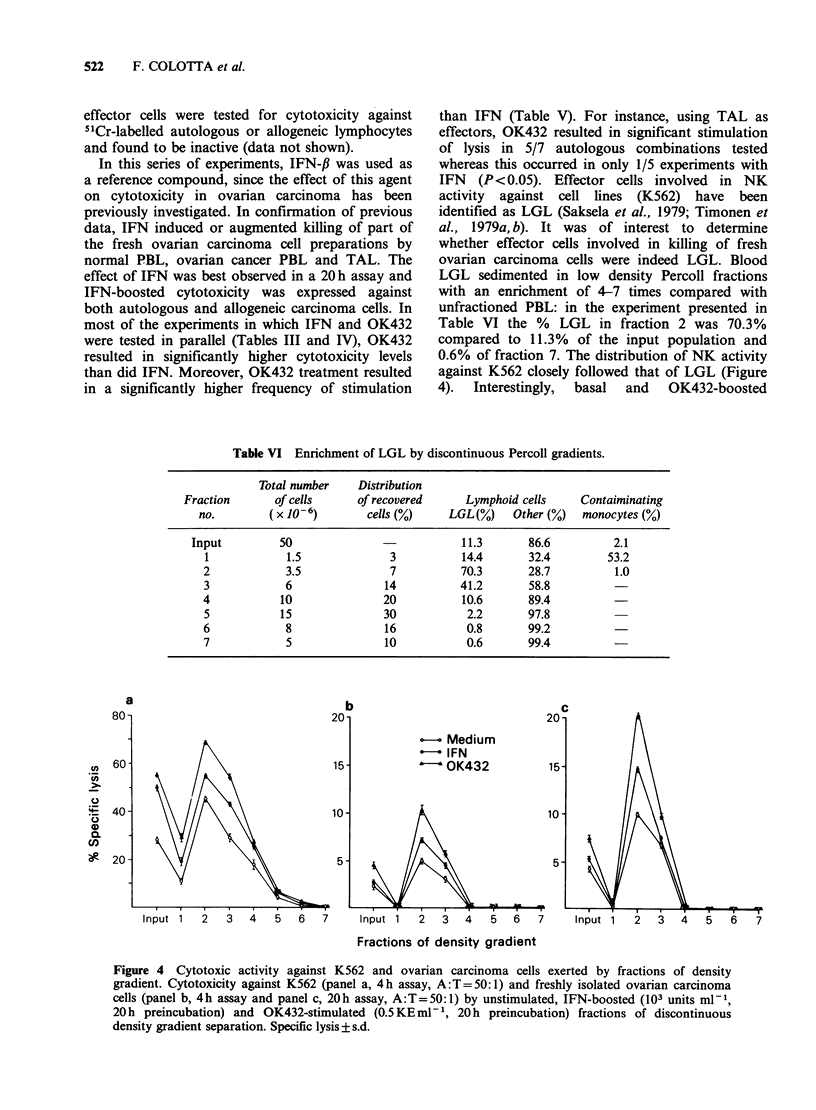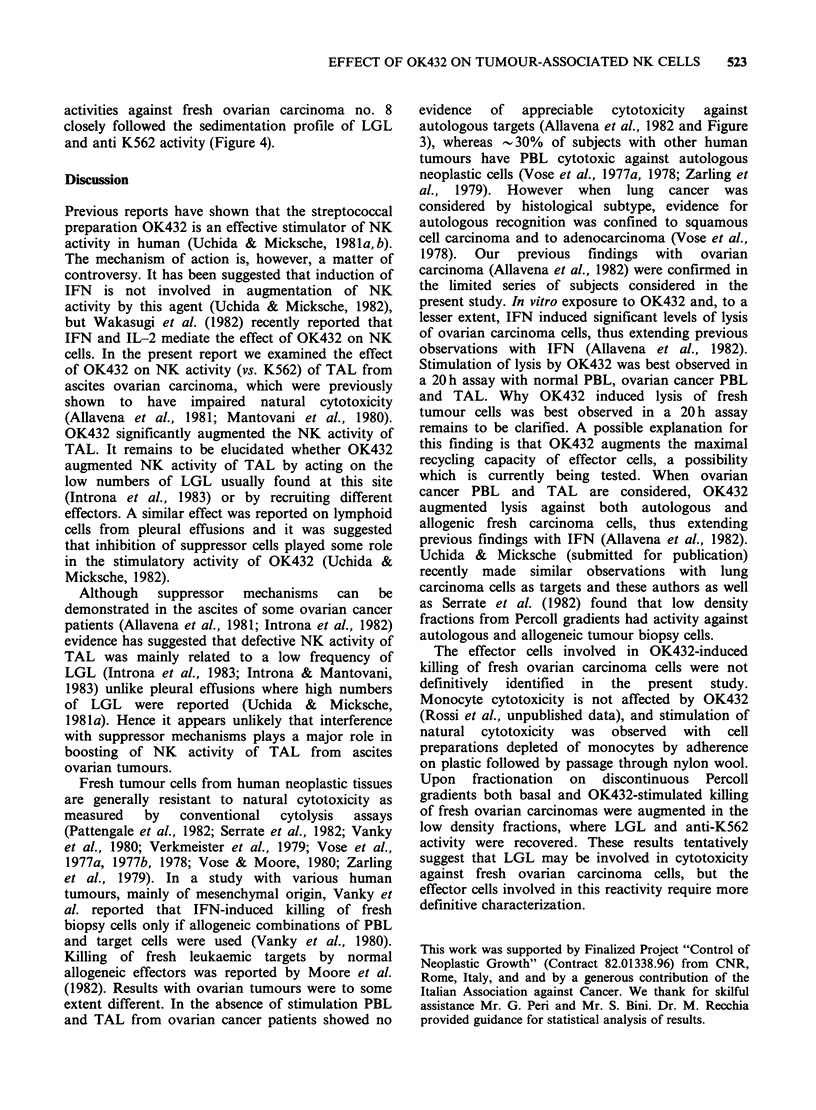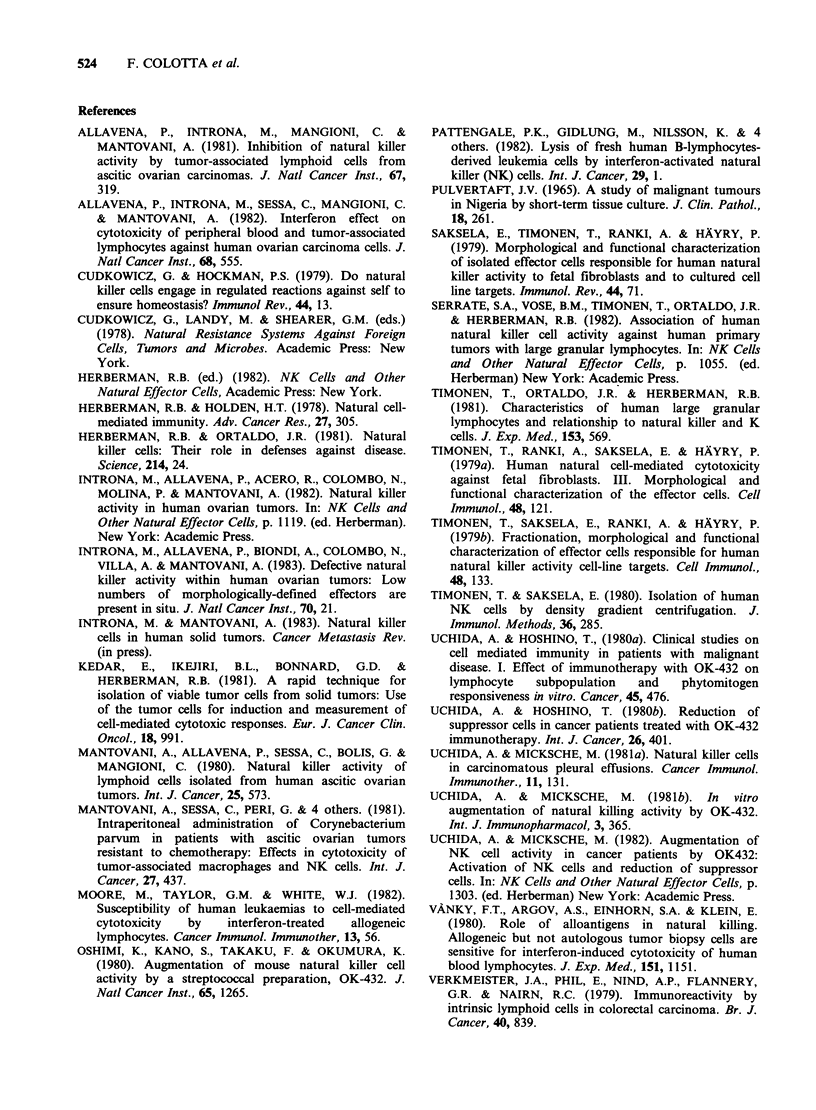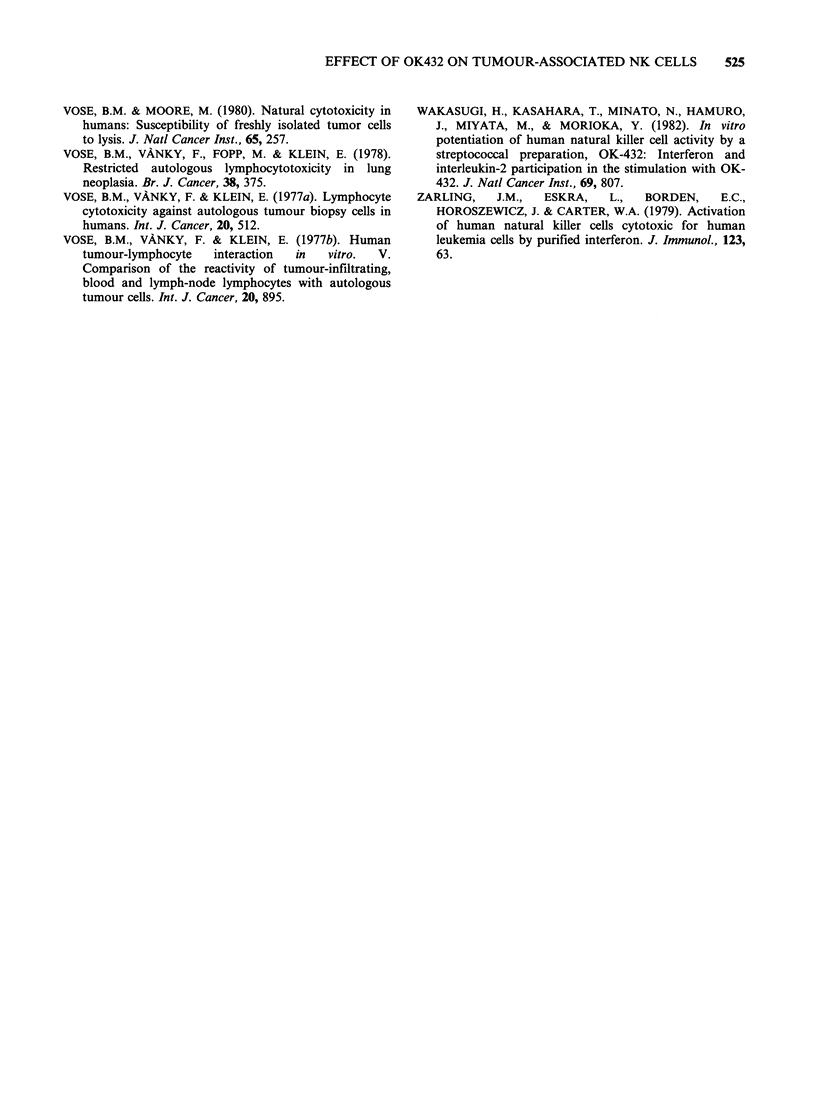# Effect of a streptococcal preparation (OK432) on natural killer activity of tumour-associated lymphoid cells in human ovarian carcinoma and on lysis of fresh ovarian tumour cells.

**DOI:** 10.1038/bjc.1983.224

**Published:** 1983-10

**Authors:** F. Colotta, A. Rambaldi, N. Colombo, L. Tabacchi, M. Introna, A. Mantovani

## Abstract

The streptococcal preparation OK432 was studied for its effects on natural killer (NK) activity of peripheral blood lymphocytes (PBL) from normal donors and from ovarian cancer patients, and of tumour-associated lymphocytes (TAL) from peritoneal effusions. OK432 augmented NK activity against the susceptible K562 line and induced killing of the relatively resistant Raji line. Freshly isolated ovarian carcinoma cells were relatively resistant to killing by unstimulated PBL and TAL. OK432 induced significant, though low, levels of cytotoxicity against 51Cr-labelled ovarian carcinoma cells. Augmentation of killing of fresh tumour cells by OK432 was best observed in a 20 h assay and both autologous and allogeneic targets were lysed. PBL were separated on discontinuous Percoll gradients. Unstimulated and OK432-boosted activity were enriched in the lower density fractions where large granular lymphocytes (LGL) and activity against K562 were found. Thus, OK432 augments NK activity of PBL and TAL in human ovarian carcinomas and induces low, but significant, levels of killing of fresh tumour cells. Effector cells involved in killing of fresh ovarian tumours copurify with LGL on discontinuous gradients of Percoll.


*Br. J. Cancer* (1983), **48**, 515–525

### Effect of a streptococcal preparation (OK432) on natural killer activity of tumour-associated lymphoid cells in human ovarian carcinoma and on lysis of fresh ovarian tumour cells

F. Colotta, A. Rambaldi, N. Colombo[1], L. Tabacchi[1], M. Introna & A. Mantovani

*Istituto di Ricerche Farmacologiche "Mario Negri" Via Eritrea, 62–20157 Milan, [1]Clinical Ostetrico-Ginecologia, Università di Milano, Ospedale S. Gerardo, Monza (Milan), Italy*

**Summary** The streptococcal preparation OK432 was studied for its effects on natural killer (NK) activity of peripheral blood lymphocytes (PBL) from normal donors and from ovarian cancer patients, and of tumour-associated lymphocytes (TAL) from peritoneal effusions. OK432 augmented NK activity against the susceptible K562 line and induced killing of the relatively resistant Raji line. Freshly isolated ovarian carcinoma cells were relatively resistant to killing by unstimulated PBL and TAL. OK432 induced significant, though low, levels of cytotoxicity against $^{51}$Cr-labelled ovarian carcinoma cells. Augmentation of killing of fresh tumour cells by OK432 was best observed in a 20 h assay and both autologous and allogeneic targets were lysed. PBL were separated on discontinuous Percoll gradients. Unstimulated and OK432-boosted activity were enriched in the lower density fractions where large granular lymphocytes (LGL) and activity against K562 were found. Thus, OK432 augments NK activity of PBL and TAL in human ovarian carcinomas and induces low, but significant, levels of killing of fresh tumour cells. Effector cells involved in killing of fresh ovarian tumours copurify with LGL on discontinuous gradients of Percoll.

Natural killer (NK) cells are defined on the basis of their ability to cause rapid lysis of susceptible target cell lines in the absence of overt sensitization (Cudkowicz *et al.*, 1978; Herberman & Holden, 1978; Herberman & Ortaldo, 1981; Herberman, 1982). A role for NK cells has been suggested in surveillance against neoplasia and in the regulation of growth of established tumours and metastasis (Cudkowicz *et al.*, 1978; Cudkowicz & Hockman, 1979; Herberman & Ortaldo, 1981). The regulation of NK activity is complex and various agents have been shown to stimulate natural cytotoxicity (Cudkowicz *et al.*, 1978; Cudkowicz & Hockman, 1979; Herberman & Holden, 1978; Herberman & Ortaldo, 1981; Herberman, 1982). OK432 is a streptococcal preparation with immunomodulatory activity (Uchida & Hoshino, 1980*a*, *b*). OK432 has been shown to augment NK activity *in vivo* or *in vitro*, in rodents and humans (Oshimi *et al.*, 1980; Uchida & Micksche, 1981,*a*, *b*). IFN and interleukin 2 (IL2) may be involved in the activation of NK cells by OK432 (Wakasugi *et al.*, 1982). Unlike cell lines, fresh tumour cells are usually relatively resistant to natural cytotoxicity by unstimulated effector cells (Moore *et al.*, 1982; Pattengale *et al.*, 1982; Serrate *et al.*, 1982; Vanky *et al.*, 1980; Verkmeister *et al.*, 1979; Vose *et al.*, 1977*a*, *b*; 1978; Vose & Moore, 1980; Zarling *et al.*, 1979). Vanky *et al.*, (1980) showed that in various human tumours IFN could induce or augment cytotoxicity against fresh neoplastic cells provided allogeneic combinations of effectors and targets were studied. As found for other human tumours, freshly isolated ovarian carcinoma cells were relatively resistant to NK activity (Allavena *et al.*, 1982; Mantovani *et al.*, 1980). *In vitro* exposure of effector cells (both allogeneic and autologous) to IFN caused modest enhancement of lysis using lymphoid cells isolated from peripheral blood and ascites tumour (Allavena *et al.*, 1982; Mantovani *et al.*, 1980).

The capacity of OK432 to stimulate NK activity prompted us to investigate the effect of this agent on natural cytotoxicity in human ovarian carcinoma, a tumour previously studied for this parameter in some detail (Allavena *et al.*, 1982; Introna *et al.*, 1983; Mantovani *et al.*, 1980; 1981). Results indicate that OK432 augments NK activity of peripheral blood (PBL) and tumour-associated lymphoid cells (TAL) in this human neoplasm. Moreover, following exposure of PBL or TAL to OK432, appreciable levels of lysis of fresh ovarian carcinoma cells were observed with both autologous or allogeneic effectors.

### Materials and methods

### *Human subjects*

Eleven patients with histologically confirmed ascites ovarian epithelial tumours admitted to the

Correspondence: F. Colotta.
Received 21 April 1983; accepted 8 July 1983.

© The Macmillan Press Ltd., 1983

Department of Oncology, Clinica Ostetrica e Ginecologica, Università di Milano, Ospedale S. Gerardo, Monza (Milan), formed the case list for this study (Table I). All patients had cancers classified as stages III and IV. In addition, in one criss-cross experiment, a patient with lymphoma was used (Patient no. 2, Table III). The presence of tumour cells in ascitic fluids was independently checked by pathologists. The control population consisted of 40 normal healthy adult volunteers. Heparinized venous blood (10–50 ml) was obtained by venipuncture, and carcinomatous ascites were collected by paracentesis or at laparotomy. Solid tumour specimens were obtained during surgery.

### *PBL*

Blood was diluted 1:5 with PBS (Eurobio, Paris), and 40 ml was then placed on 10 ml Ficoll-Hypaque (Eurobio) for centrifugation at 400 *g* for 20 min at room temperature. Mononuclear cells were collected at the interface and washed with PBS, and $10–30 \times 10^6$ cells were incubated in 10–20 ml of RPMI-1640 medium supplemented with 20% FBS (Gibco–Biocult, Glasgow, Scotland) for 45 min at 37°C in plastic petri dishes (≠3003; Falcon Plastics, Oxnard, Calif.). Nonadherent cells were collected, centrifuged at 400 *g* for 5 min, and finally resuspended in RPMI-1640 medium with 10% FBS and 50 $\mu$g gentamycin $ml^{-1}$. Nonadherent cell preparations were only partially depleted of monocytes inasmuch as 2–4% of these cells as compared to 10–30% of the original mononuclear cell suspensions (Mantovani *et al.*, 1980) were mononuclear phagocytes as assessed by morphology, avid uptake of neutral red, and staining for nonspecific esterase.

### *TAL and ovarian carcinoma cells*

Enriched preparations of ovarian tumor cells and TAL were obtained by stepwise application of density and velocity sedimentation on discontinuous Ficoll–Hypaque gradient (Mantovani *et al.*, 1980).

Briefly, Ficoll–Hypaque separated ascites mononuclear cells, deprived of macrophages by adherence, were fractionated on discontinuous Ficoll–Hypaque (10 ml 75%, 15 ml 100% gradient). Tumour cells sedimented on top of the 75% fraction and lymphoid cells were found at the 75–100% interface. Only preparations of >95% purity assessed morphologically were used.

For the isolation of ovarian cells and tumour-associated lymphocytes from solid tumours, specimens were minced mechanically and then exposed for 45 min at 37°C to 0.3% collagenase (#40130; Sigma Chemical Co., St. Louis, Mo., 02 Worthington Biomedical Corp., Freehold, N.J.) in BME (Eurobio) containing 10 $\mu$g DNase $ml^{-1}$. After 2 washes with 50 ml BME, disaggregated cells were then treated as described above for the cellular preparations isolated from the ascitic fluids.

### *IFN*

Partially purified human fibroblast IFN was obtained from Serono (Frone, Serono, Rome Italy). Lymphoid cells ($1–5 \times 10^6$ $ml^{-1}$) were cultured for 20 h in the presence of $10^3$ U IFN $ml^{-1}$ in growth medium. Control lymphocytes were cultured alone. Lymphoid cells were washed with 50 ml PBS before their cytotoxic capacity was tested.

### *OK432*

OK432, a lyophilized preparation of attenuated strain Su of Streptococcus haemoliticus (Group A, Type 3), was obtained from Chugai Pharmaceutical Co. Ltd., Tokyo, Japan. Effector cells at a concentration of $1–2 \times 10^6$ $ml^{-1}$ in RPMI 1640 10% FBS (Gibco) were cultured for 20 h with OK432 (usually 0.5 KE $ml^{-1}$, 1 KE = 0.1 mg dried streptococci) in humidified atmosphere of 5% $CO_2$ in air at 37°C. Lyophilized powder was reconstituted in PBS. Control lymphocytes were cultured alone. After incubation lymphoid cells were washed 2–3 times and resuspended in complete medium.

### *Target cells*

NK activity of PBL was measured against the highly sensitive K562 (Allavena *et al.*, 1981) and relatively resistant Raji cell lines (Pulvertaft, 1965). Target cells were pre-incubated with 20–50 $\mu$Ci $^{51}Cr$ (Radiochemical Centre, Amersham, Buckinghamshire, England) at 37°C for 45 min.

Fresh ovarian carcinoma cells ($1–5 \times 10^6$ in 0.2–1 ml growth medium) were labelled by incubation for 20 h at 37°C (overnight) with 20–50 $\mu$Ci $^{51}Cr$ (Kedar *et al.*, 1981).

Labelled cells were washed twice with 50 ml of PBS before use in cytolysis assays.

### *Cytotoxicity assay*

$^{51}Cr$ labelled tumour cells ($10^4$) were cultured in 0.1 ml RPMI 1640 medium with 10% serum in round-bottomed wells of Microtest plates (Sterilin Ltd., Teddington, Middlesex, England). The routinely employed attacker:target (A:T) ratios were 25:1 and 50:1 for ovarian carcinoma cells. Lysis of K562 and Raji was routinely measured at A:T ranging from 6:1 to 50:1, but only data for 25:1 (K562) and 50:1 (Raji) are presented. Isotope release was determined after 4 and 20 h of incubation, except for occasional experiments in which cytotoxicity was measured only at 4 h.

Isotope release was calculated as (A/B) × 100, were A is the isotope in the supernatant and B is the total incorporated radioactivity released by incubation with 1% sodium dodecyl sulfate in water. Specific lysis was calculated by substraction of the spontaneous isotope release of tumour cells alone. Spontaneous $^{51}Cr$ release from ovarian carcinoma cells ranged from 8.6 to 30.6% after 4 h incubation and from 21.6 to 37.4% after 20 h (Table I). In 4 experiments, control cultures to which unlabelled ovarian carcinoma cells were added instead of lymphocytes, were included; isotope release was not different from that of labelled target cells alone.

*Percoll fractionation of peripheral blood lymphocytes*

Lymphoid cells were separated on Percoll as described in detail elsewhere (Introna *et al.*, 1983; Timonen & Saksela, 1980; Timonen *et al.*, 1981). Briefly, discontinuous gradients of Percoll (7 steps) from 42.5% to 55% (v/v) were prepared with RPMI 1640 medium with 10% FBS, after adjusting the osmolarity to 285 mosmol. Cells were deprived of monocytes by adherence on plastic, carbonyl iron and nylon wool columns and kept at 4°C overnight before fractionation at 550 *g* for 30 min.

Enrichment in large granular lymphocytes (LGL) was evaluated on May–Grunwald–Giemsa stained cytocentrifuge smears (Introna *et al.*, 1983; Timonen & Saksela, 1980; Timonen *et al.*, 1981).

*Statistical analysis*

At least 3 replicates per experimental group were employed throughout, and results of cytotoxicity tests were calculated as mean ± s.d.; 4–6% increases in isotope release above base line were usually statistically significant at $P<0.05$ (Duncan's new multiple-range test). The incidence of stimulation was analysed by Fisher's exact test.

**Results**

A first series of experiments was designed to define the conditions for optimal augmentation of normal PBL NK activity against cell lines (K562 and Raji) by OK432. Fibroblast IFN was used as a standard immunostimulatory agent. In agreement with previous reports OK432 augmented lysis of the NK susceptible K562 target (Figure 1) and induced killing of the relatively resistant Raji line (Figure 2). Optimal stimulation was observed at OK432 concentrations of 0.1–0.5 KE $ml^{-1}$ and after incubation times of 20 h (data not shown). Therefore in subsequent experiments effector cells were exposed to 0.5 KE $ml^{-1}$ for 20 h. Previous studies have shown that TAL from ascites and, to an even greater extent, solid ovarian tumours have (Allavena *et al.*, 1981) usually low NK activity compared to PBL effectors (Allavena *et al.*, 1982). It was therefore of interest to elucidate whether OK432 stimulated NK activity (*vs.* K562) of TAL and, by way of comparison, of PBL from ovarian cancer patients. As shown by the representative experiment in Table II, OK432 was effective at stimulating NK activity of PBL and TAL and,

**Table II** Effect of OK432 on NK activity *vs* K562 of PBL and TAL from ovarian carcinoma patients

| | *% specific lysis (± s.d.) with* | | |
|---|---|---|---|
| *Effector cells*[a, b] | *medium* | *IFN* | *OK432* |
| Normal PBL | 34.3 ± 4.0 | 41.2 ± 2.9 | 47.4 ± 1.1 |
| Patient's PBL (no. 8) | 18.9 ± 0.2 | 25.6 ± 0.1 | 33.5 ± 4.0 |
| Patient's TAL (no. 8) | 6.5 ± 0.8 | NT | 54.7 ± 1.2 |

[a]Effector cells were incubated for 20 h with IFN-$\beta$ ($10^3$ units $ml^{-1}$) or OK432 (0.5 KE $ml^{-1}$).

[b]A:T = 50:1.

NT: not tested.

**Table I** Characteristics of ovarian carcinoma cells used as targets in cytolysis assays

| *Patient no.* | *Tumour* | *Histology* | *Grade* | *Stage* | *Spontaneous release (%)* 4 h | *Spontaneous release (%)* 20 h |
|---|---|---|---|---|---|---|
| 1 | Solid | undifferentiated | 3 | III | 12.6 | 36.2 |
| 3 | Ascites | serous | 3 | III | 13.8 | 21.6 |
| 4 | Ascites | undifferentiated | 2 | IV | 9.9 | 32.3 |
| 5 | Solid | serous | 2 | IV | 15.4 | 33.3 |
| 6 | Ascites | serous | 3 | III | 13.5 | 34.5 |
| 7 | Ascites | serous | 1 | IV | 8.6 | 33.8 |
| 8 | Ascites | serous | 1 | IV | 11.3 | 36.6 |
| 9 | Ascites | undifferentiated | 4 | III | 10.5 | 29.9 |
| 10 | Ascites | serous | 3 | III | 30.6 | 37.4 |
| 11 | Ascites | serous | 2 | III | 18.3 | NT |
| 12 | Ascites | serous | 1 | IV | 12.2 | NT |

NT: not tested.

**Figure 1** Effect of OK-432 on NK activity *vs* K562 cells. Panel a shows boosting of activity by OK-432 (0.5 KE ml$^{-1}$ for 20 h) and by IFN ($10^3$ Units ml$^{-1}$ for 20 h) tested at different A:T ratios. Panels b and c show a typical dose-response experiment (A:T = 25:1) using OK432 (panel B) and IFN (C). Specific lysis $\pm$ s.d.

when IFN was tested in parallel, levels of boosting were somewhat higher with OK432 than those with IFN. Similar results were obtained in 5 additional patients tested for boosting of NK activity (*vs.* K562) by OK432. We then examined the capacity of OK432 to stimulate killing of fresh ovarian carcinoma cells, using PBL from normal donors as effectors or PBL and TAL from cancer patients in autologous or allogeneic combinations. In agreement with previous results in a larger series of subjects, (Allavena *et al.*, 1981; Mantovani *et al.*, 1980), PBL and TAL from ovarian cancer patients showed little or no cytotoxicity against autologous or allogeneic carcinoma, with levels of activity (usually $\leqslant$10% specific lysis at 4 h and <15% at 20 h), when present, not exceeding those occasionally encountered with normal PBL (Figure 3). Table III presents data on the effect of OK432, and by way of comparison IFN, on killing of fresh ovarian carcinoma cells. Only in a minority of the experiments did we have available lymphoid and tumour cells from 2 different patients on the same day so as to run criss-cross combinations of effectors and target cells: these experiments are also shown in detail in Table III, while Table IV presents all experiments performed and Table V sets out a synopsis of overall effect of OK432 on killing of fresh ovarian carcinoma cells. OK432 was able to augment cytotoxicity against fresh ovarian tumours with all effector populations tested (normal PBL, ovarian cancer PBL and TAL). OK432-induced cytotoxicity was best observed when a 20 h assay was performed. Boosting of cytotoxicity was rarely observed, particularly with PBL and TAL from ovarian cancer patients in a 4 h assay (only in 2/8 cases with PBL and 1/9 with TAL) with low levels of stimulation (absolute increases in lysis, 11.0% and 9.6% for PBL and 7.2% for TAL). Augmentation of killing of fresh ovarian tumours was best evident in a 20 h $^{51}Cr$ release assay, both in terms of the frequency of positive responses (for instance for TAL 5/7 at 20 h compared to 1/9 at 4 h) and in terms of absolute levels of specific lysis attained (Table IV). OK432 augmented the cytotoxic activity of PBL and TAL against both autologous and allogeneic target cells. For instance in the experiments shown in Table III OK432-stimulated TAL from Patients No. 4 caused 13.3 $\pm$ 1.7% lysis of autologous and 7.4 $\pm$ 0.2% lysis of allogeneic carcinoma cells with absolute increases in cytotoxicity of 10.4 and 7.5% respectively ($P$<0.01). In one experiment OK432-boosted

**Figure 2** Effect of OK432 on natural cytotoxicity against Raji cells. Panel a (4 h assay) and b (20 h assay) show boosting of activity by OK432 (0.5 KE ml$^{-1}$, 20 h preincubation) and by IFN ($10^3$ Units ml$^{-1}$, 20 h preincubation) tested at different A:T ratios. Panels c and d (4 h and 20 h assay, OK432), e and f (4 h and 20 h assay, IFN) show typical dose-response experiments (A:T = 50:1). Specific lysis ±s.d.

**Figure 3** Cytotoxicity of PBL and TAL against autologous and allogeneic freshly isolated carcinoma cells. Cytolysis was measured in a 4 h (●) and a 20 h (▲) assay, with the use of an A:T = 50:1.

**Table III** Effect of OK-432 on cytotoxicity of PBL and TAL against autologous or allogeneic carcinoma cells

| | | | Specific lysis[b] (%) | | | | | |
|---|---|---|---|---|---|---|---|---|
| | | | 4 h | | | 20 h | | |
| *Experiment* | *Target cells* | *Effector cells*[a] | *Medium* | *IFN* ($10^3$ U ml$^{-1}$) | *OK-432* (0.5 K.E. ml$^{-1}$) | *Medium* | *IFN* ($10^3$ U ml$^{-1}$) | *OK-432* (0.5 KE ml$^{-1}$) |
| 1 | Patient no. 3 | Normal PBL | 0.2±0.3 | 4.2±0.2 | 3.6±0.2 | 1.1±2.2 | 5.0±2.2 | 13.4±3.6[d,f] |
| | | Patient no. 3 PBL | 1.6±0.2 | 2.2±0.3 | 2.4±0.8 | 2.5±1.4 | 4.4±0.9 | 9.6±0.9[d,f] |
| | | TAL | 2.6±0.2 | 3.0±0.8 | 2.2±0.4 | 1.7±1.0 | 4.0±1.6 | 5.0±1.5[c] |
| | | Patient no. 4 TAL | 0.5±0.6 | 2.5±0.1 | 2.6±0.1 | −0.1±0.7 | 1.2±0.4 | 7.4±0.2[d,f] |
| | Patient no. 4 | Normal PBL | 15.8±1.6 | 34.0±1.7[d] | 31.4±1.1[d] | 24.4±2.4 | 29.4±4.0 | 31.2±0.4[c] |
| | | Patient no. 4 PBL | 7.6±1.3 | 18.8±0.1[d] | 17.2±0.6[d] | 15.4±1.1 | 19.3±2.2 | 26.7±2.3[d,e] |
| | | TAL | 1.8±0.5 | 5.2±0.6 | 4.4±1.0 | 2.9±1.2 | 4.1±0.7 | 13.3±1.7[d,f] |
| | | Patient no. 3 TAL | 5.4±0.9 | 21.2±1.3[d] | 22.0±1.6[d] | 9.9±1.5 | 23.7±1.0[d] | 25.0±3.1[d] |
| 2 | Patient no. 5 | Normal PBL | 0.7±1.0 | 9.5±0.8[d] | 12.4±0.4[d] | 6.6±2.5 | 21.1±1.0[d] | 29.9±1.5[d,e] |
| | | Patient no. 6 TAL | 0.7±0.4 | 2.8±0.8 | 12.8±1.3[d,f] | 5.3±0.2 | 10.3±1.1[d] | 27.6±1.5[d,f] |
| | Patient no. 6 | Normal PBL | −3.6±2.0 | 4.2±1.6[c] | 6.2±0.8[d] | −2.1±0.8 | 7.2±1.7[d] | 9.8±6.9[d] |
| | | Patient no. 6 TAL | 1.3±0.9 | 1.6±0.7 | 4.7±1.0 | −1.8±2.2 | −1.0±0.7 | 9.3±4.1[d,f] |
| 3 | Patient no. 1 | Normal PBL | 1.4±2.0 | 1.6±1.2 | 1.0±0.8 | 4.3±0.7 | 6.6±1.5 | 10.6±2.6[c] |
| | | Patient no. 1 PBL | 2.5±2.3 | 1.6±0.8 | 1.7±2.4 | 4.7±2.7 | 5.8±1.6 | 5.0±1.9 |
| | | TAL | 0.8±1.0 | 1.5±1.4 | 2.8±2.6 | 1.8±1.5 | 8.9±1.3[d] | 14.0±1.3[d] |
| | | Patient no. 2 TAL | 1.9±2.2 | 2.2±0.8 | 1.0±0.9 | 3.0±3.5 | 2.7±2.0 | 6.0±2.8 |
| | Patient no. 2 | Normal PBL | −1.0±0.4 | 1.7±0.9 | 2.1±1.0 | 0.4±0.5 | 2.8±1.3 | 29.0±2.7[d,f] |
| | | Patient no. 2 TAL | −0.4±1.0 | 2.8±1.3 | 29.0±2.7[d,f] | −1.5±0.6 | 0.2±1.6 | 18.0±2.1[d,f] |
| | | Patient no. 1 PBL | 0.6±1.4 | 2.0±1.3 | 6.1±0.4[c] | −0.8±2.1 | 2.0±1.7 | 21.6±4.0[d,f] |
| | | TAL | 0.3±0.8 | 1.5±0.3 | −1.1.±2.0 | 3.0±3.5 | 2.7±2.0 | 6.0±2.8 |

[a]A:T = 50:1.

[b]Mean ± s.d.

[c]$P < 0.05$ *vs* unstimulated cells.

[d]$P < 0.01$ *vs* unstimulated cells.

[e]$P < 0.05$ *vs* IFN-boosted cells.

[f]$P < 0.01$ *vs* IFN-boosted cells.

**Table IV** Effect of OK-432 on cytotoxicity against fresh ovarian carcinoma cells

| *Ovarian tumour no.* | *Effector cells*[a] | *% specific lysis with medium/with IFN/with OK-432* 4 *h* | 20 *h* |
|---|---|---|---|
| 1 | Normal PBL | 1.4/1.6/1.0 | 4.3/6.6/10.6[b] |
| | Autologous PBL | 2.5/1.6/1.7 | 4.7/5.8/5.0 |
| | TAL | 0.8/1.5/2.8 | 1.8/8.9[c]/14.0[c,d] |
| | Patient no. 2 TAL | 1.9/2.2/1.0 | 3.0/2.7/6.0 |
| 3 | Normal PBL | 0.2/4.2/3.6 | 1.1/5.0/13.4[c,d] |
| | Autologous PBL | 1.6/2.2/2.4 | 2.5/4.4/9.6[b,d] |
| | TAL | 2.6/3.0/2.2 | 1.7/4.0/5.0 |
| | Patient no. 4 TAL | 0.5/2.5/2.6 | −0.1/1.2/7.4[b,d] |
| 4 | Normal PBL | 15.8/34.0[c]/31.4[c] | 24.4/29.4[b]/31.2[b] |
| | Autologous PBL | 7.6/18.8[c]/17.2[c] | 15.4/19.3/26.7[b,e] |
| | TAL | 1.8/5.2/4.4 | 2.9/4.1/13.3[c,e] |
| | Patient no. 3 TAL | 5.4/21.2[c]/22.0[c] | 9.9/23.7[c]/25.0[c] |
| 5 | Normal PBL | 0.7/9.5[c]/12.4[c] | 6.6/21.1[c]/29.9[c,d] |
| | Patient no. 6 TAL | 0.7/2.8/12.8[c,e] | 5.3/10.3[b]/27.6[c,e] |
| 6 | Normal PBL | −3.6/4.2[b]/6.2[b] | −2.1/7.2[b]/9.8[c] |
| | Autologous TAL | 1.3/1.6/4.7 | −1.8/−1.0/9.3[c,e] |
| 7 | Normal PBL | 1.3/NT/2.3 | 6.2/NT/14.2[c] |
| | Autologous PBL | 0/NT/0 | −2.7/NT/5.8[b] |
| | TAL | −0.1/NT/0.3 | −6.4/NT/5.6[b] |
| 8 | Normal PBL | −1.7/NT/0.5 | −6.6/NT/8.5[c] |
| | Autologous PBL | 0.7/NT/−1.4 | −10.1/NT/4.1[c] |
| 9 | Normal PBL | 0.8/1.7/10.1[c] | 2.4/5.6/32.7[c,e] |
| | Autologous PBL | 0.9/0.2/4.3 | 3.2/0.9/23.8[c,e] |
| | TAL | 0.6/NT/7.8[b] | 4.3/NT/28.4[c] |
| 10 | Normal PBL | 4.1/15.4[c]/23.9[c,d] | 8.9/25.2[c]/27.9[c] |
| | Autologous PBL | 0.2/4.5/11.2[c,d] | −0.1/5.4[d]/22.8[c,e] |
| | TAL | −1.3/−1.3/−0.4 | −2.9/0.3/2.3 |
| 11 | Normal PBL | −1.2/NT/0.1 | NT |
| | Autologous PBL | −0.7/NT/0.3 | NT |
| | TAL | −1.6/NT/0.8 | NT |
| 12 | Autologous TAL | 8/NT/10.9 | NT |

[a]A:T = 50:1.
[b]$P < 0.05$ *vs* unstimulated cells.
[c]$P < 0.01$ *vs* unstimulated cells.
[d]$P < 0.005$ *vs* IFN-boosted cells.
[e]$P < 0.01$ *vs* IFN-boosted cells.
NT = not tested.

**Table V** Augmentation of lysis of fresh ovarian carcinoma cells by OK-432: Summary of overall results.

| *Effector cells* | *Incubation time (h)* | *No. tumour cells with enhanced lysis/total tested* with IFN | with OK-432 | *Absolute increase in lysis (% median with range)* with IFN | with OK-432 |
|---|---|---|---|---|---|
| Normal PBL | 4 | 5/7 | 5/10 | 8.8(4.0–18.2) | 11.7(9.3–19.8) |
| | 20 | 4/7 | 9/9[a] | 11.9(5.0–16.3) | 13.7(6.3–30.3) |
| Patient PBL | 4 | 2/5 | 2/8 | 4.3; 11.2 | 9.6; 11.0 |
| (autologous) | 20 | 1/5 | 6/7[a] | 5.5 | 12.7(7.1–22.9) |
| Patient TAL | 4 | 0/5 | 1/9 | — | 7.2 |
| (autologous) | 20 | 1/5 | 5/7[a] | 7.1 | 12.2(10.4–24.1) |
| Patient TAL | 4 | 1/4 | 1/4 | 15.8 | 16.6 |
| (allogeneic) | 20 | 3/4 | 3/4 | 5.6(5.0–18.8) | 18.0(7.5–22.3) |

[a]$P < 0.05$ (Fischer's exact test) *vs* IFN-boosted cells.

effector cells were tested for cytotoxicity against $^{51}Cr$-labelled autologous or allogeneic lymphocytes and found to be inactive (data not shown).

In this series of experiments, IFN-$\beta$ was used as a reference compound, since the effect of this agent on cytotoxicity in ovarian carcinoma has been previously investigated. In confirmation of previous data, IFN induced or augmented killing of part of the fresh ovarian carcinoma cell preparations by normal PBL, ovarian cancer PBL and TAL. The effect of IFN was best observed in a 20 h assay and IFN-boosted cytotoxicity was expressed against both autologous and allogeneic carcinoma cells. In most of the experiments in which IFN and OK432 were tested in parallel (Tables III and IV), OK432 resulted in significantly higher cytotoxicity levels than did IFN. Moreover, OK432 treatment resulted in a significantly higher frequency of stimulation than IFN (Table V). For instance, using TAL as effectors, OK432 resulted in significant stimulation of lysis in 5/7 autologous combinations tested whereas this occurred in only 1/5 experiments with IFN ($P<0.05$). Effector cells involved in NK activity against cell lines (K562) have been identified as LGL (Saksela *et al.*, 1979; Timonen *et al.*, 1979*a,b*). It was of interest to determine whether effector cells involved in killing of fresh ovarian carcinoma cells were indeed LGL. Blood LGL sedimented in low density Percoll fractions with an enrichment of 4–7 times compared with unfractioned PBL: in the experiment presented in Table VI the % LGL in fraction 2 was 70.3% compared to 11.3% of the input population and 0.6% of fraction 7. The distribution of NK activity against K562 closely followed that of LGL (Figure 4). Interestingly, basal and OK432-boosted

**Table VI** Enrichment of LGL by discontinuous Percoll gradients.

| *Fraction no.* | *Total number of cells* ($\times 10^{-6}$) | *Distribution of recovered cells* (%) | *Lymphoid cells* *LGL* (%) | *Lymphoid cells* *Other* (%) | *Containinating monocytes* (%) |
|---|---|---|---|---|---|
| Input | 50 | — | 11.3 | 86.6 | 2.1 |
| 1 | 1.5 | 3 | 14.4 | 32.4 | 53.2 |
| 2 | 3.5 | 7 | 70.3 | 28.7 | 1.0 |
| 3 | 6 | 14 | 41.2 | 58.8 | — |
| 4 | 10 | 20 | 10.6 | 89.4 | — |
| 5 | 15 | 30 | 2.2 | 97.8 | — |
| 6 | 8 | 16 | 0.8 | 99.2 | — |
| 7 | 5 | 10 | 0.6 | 99.4 | — |

**Figure 4** Cytotoxic activity against K562 and ovarian carcinoma cells exerted by fractions of density gradient. Cytotoxicity against K562 (panel a, 4 h assay, A:T = 50:1) and freshly isolated ovarian carcinoma cells (panel b, 4 h assay and panel c, 20 h assay, A:T = 50:1) by unstimulated, IFN-boosted ($10^3$ units $ml^{-1}$, 20 h preincubation) and OK432-stimulated (0.5 KE $ml^{-1}$, 20 h preincubation) fractions of discontinuous density gradient separation. Specific lysis ± s.d.

activities against fresh ovarian carcinoma no. 8 closely followed the sedimentation profile of LGL and anti K562 activity (Figure 4).

### Discussion

Previous reports have shown that the streptococcal preparation OK432 is an effective stimulator of NK activity in human (Uchida & Micksche, 1981*a,b*). The mechanism of action is, however, a matter of controversy. It has been suggested that induction of IFN is not involved in augmentation of NK activity by this agent (Uchida & Micksche, 1982), but Wakasugi *et al.* (1982) recently reported that IFN and IL–2 mediate the effect of OK432 on NK cells. In the present report we examined the effect of OK432 on NK activity (*vs.* K562) of TAL from ascites ovarian carcinoma, which were previously shown to have impaired natural cytotoxicity (Allavena *et al.*, 1981; Mantovani *et al.*, 1980). OK432 significantly augmented the NK activity of TAL. It remains to be elucidated whether OK432 augmented NK activity of TAL by acting on the low numbers of LGL usually found at this site (Introna *et al.*, 1983) or by recruiting different effectors. A similar effect was reported on lymphoid cells from pleural effusions and it was suggested that inhibition of suppressor cells played some role in the stimulatory activity of OK432 (Uchida & Micksche, 1982).

Although suppressor mechanisms can be demonstrated in the ascites of some ovarian cancer patients (Allavena *et al.*, 1981; Introna *et al.*, 1982) evidence has suggested that defective NK activity of TAL was mainly related to a low frequency of LGL (Introna *et al.*, 1983; Introna & Mantovani, 1983) unlike pleural effusions where high numbers of LGL were reported (Uchida & Micksche, 1981*a*). Hence it appears unlikely that interference with suppressor mechanisms plays a major role in boosting of NK activity of TAL from ascites ovarian tumours.

Fresh tumour cells from human neoplastic tissues are generally resistant to natural cytotoxicity as measured by conventional cytolysis assays (Pattengale *et al.*, 1982; Serrate *et al.*, 1982; Vanky *et al.*, 1980; Verkmeister *et al.*, 1979; Vose *et al.*, 1977*a*, 1977*b*, 1978; Vose & Moore, 1980; Zarling *et al.*, 1979). In a study with various human tumours, mainly of mesenchymal origin, Vanky *et al.* reported that IFN-induced killing of fresh biopsy cells only if allogeneic combinations of PBL and target cells were used (Vanky *et al.*, 1980). Killing of fresh leukaemic targets by normal allogeneic effectors was reported by Moore *et al.* (1982). Results with ovarian tumours were to some extent different. In the absence of stimulation PBL and TAL from ovarian cancer patients showed no evidence of appreciable cytotoxicity against autologous targets (Allavena *et al.*, 1982 and Figure 3), whereas ~30% of subjects with other human tumours have PBL cytotoxic against autologous neoplastic cells (Vose *et al.*, 1977*a*, 1978; Zarling *et al.*, 1979). However when lung cancer was considered by histological subtype, evidence for autologous recognition was confined to squamous cell carcinoma and to adenocarcinoma (Vose *et al.*, 1978). Our previous findings with ovarian carcinoma (Allavena *et al.*, 1982) were confirmed in the limited series of subjects considered in the present study. *In vitro* exposure to OK432 and, to a lesser extent, IFN induced significant levels of lysis of ovarian carcinoma cells, thus extending previous observations with IFN (Allavena *et al.*, 1982). Stimulation of lysis by OK432 was best observed in a 20 h assay with normal PBL, ovarian cancer PBL and TAL. Why OK432 induced lysis of fresh tumour cells was best observed in a 20 h assay remains to be clarified. A possible explanation for this finding is that OK432 augments the maximal recycling capacity of effector cells, a possibility which is currently being tested. When ovarian cancer PBL and TAL are considered, OK432 augmented lysis against both autologous and allogenic fresh carcinoma cells, thus extending previous findings with IFN (Allavena *et al.*, 1982). Uchida & Micksche (submitted for publication) recently made similar observations with lung carcinoma cells as targets and these authors as well as Serrate *et al.* (1982) found that low density fractions from Percoll gradients had activity against autologous and allogeneic tumour biopsy cells.

The effector cells involved in OK432-induced killing of fresh ovarian carcinoma cells were not definitively identified in the present study. Monocyte cytotoxicity is not affected by OK432 (Rossi *et al.*, unpublished data), and stimulation of natural cytotoxicity was observed with cell preparations depleted of monocytes by adherence on plastic followed by passage through nylon wool. Upon fractionation on discontinuous Percoll gradients both basal and OK432-stimulated killing of fresh ovarian carcinomas were augmented in the low density fractions, where LGL and anti-K562 activity were recovered. These results tentatively suggest that LGL may be involved in cytotoxicity against fresh ovarian carcinoma cells, but the effector cells involved in this reactivity require more definitive characterization.

This work was supported by Finalized Project "Control of Neoplastic Growth" (Contract 82.01338.96) from CNR, Rome, Italy, and and by a generous contribution of the Italian Association against Cancer. We thank for skilful assistance Mr. G. Peri and Mr. S. Bini. Dr. M. Recchia provided guidance for statistical analysis of results.

### References

ALLAVENA, P., INTRONA, M., MANGIONI, C. & MANTOVANI, A. (1981). Inhibition of natural killer activity by tumor-associated lymphoid cells from ascitic ovarian carcinomas. *J. Natl Cancer Inst.*, **67**, 319.

ALLAVENA, P., INTRONA, M., SESSA, C., MANGIONI, C. & MANTOVANI, A. (1982). Interferon effect on cytotoxicity of peripheral blood and tumor-associated lymphocytes against human ovarian carcinoma cells. *J. Natl Cancer Inst.*, **68**, 555.

CUDKOWICZ, G. & HOCKMAN, P.S. (1979). Do natural killer cells engage in regulated reactions against self to ensure homeostasis? *Immunol Rev.*, **44**, 13.

CUDKOWICZ, G., LANDY, M. & SHEARER, G.M. (eds.) (1978). *Natural Resistance Systems Against Foreign Cells, Tumors and Microbes*. Academic Press: New York.

HERBERMAN, R.B. (ed.) (1982). *NK Cells and Other Natural Effector Cells*, Academic Press: New York.

HERBERMAN, R.B. & HOLDEN, H.T. (1978). Natural cell-mediated immunity. *Adv. Cancer Res.*, **27**, 305.

HERBERMAN, R.B. & ORTALDO, J.R. (1981). Natural killer cells: Their role in defenses against disease. *Science*, **214**, 24.

INTRONA, M., ALLAVENA, P., ACERO, R., COLOMBO, N., MOLINA, P. & MANTOVANI, A. (1982). Natural killer activity in human ovarian tumors. In: *NK Cells and Other Natural Effector Cells*, p. 1119. (ed. Herberman). New York: Academic Press.

INTRONA, M., ALLAVENA, P., BIONDI, A., COLOMBO, N., VILLA, A. & MANTOVANI, A. (1983). Defective natural killer activity within human ovarian tumors: Low numbers of morphologically-defined effectors are present in situ. *J. Natl Cancer Inst.*, **70**, 21.

INTRONA, M. & MANTOVANI, A. (1983). Natural killer cells in human solid tumors. *Cancer Metastasis Rev.* (in press).

KEDAR, E., IKEJIRI, B.L., BONNARD, G.D. & HERBERMAN, R.B. (1981). A rapid technique for isolation of viable tumor cells from solid tumors: Use of the tumor cells for induction and measurement of cell-mediated cytotoxic responses. *Eur. J. Cancer Clin. Oncol.*, **18**, 991.

MANTOVANI, A., ALLAVENA, P., SESSA, C., BOLIS, G. & MANGIONI, C. (1980). Natural killer activity of lymphoid cells isolated from human ascitic ovarian tumors. *Int. J. Cancer*, **25**, 573.

MANTOVANI, A., SESSA, C., PERI, G. & 4 others. (1981). Intraperitoneal administration of Corynebacterium parvum in patients with ascitic ovarian tumors resistant to chemotherapy: Effects in cytotoxicity of tumor-associated macrophages and NK cells. *Int. J. Cancer*, **27**, 437.

MOORE, M., TAYLOR, G.M. & WHITE, W.J. (1982). Susceptibility of human leukaemias to cell-mediated cytotoxicity by interferon-treated allogeneic lymphocytes. *Cancer Immunol. Immunother*, **13**, 56.

OSHIMI, K., KANO, S., TAKAKU, F. & OKUMURA, K. (1980). Augmentation of mouse natural killer cell activity by a streptococcal preparation, OK-432. *J. Natl Cancer Inst.*, **65**, 1265.

PATTENGALE, P.K., GIDLUNG, M., NILSSON, K. & 4 others. (1982). Lysis of fresh human B-lymphocytes-derived leukemia cells by interferon-activated natural killer (NK) cells. *Int. J. Cancer*, **29**, 1.

PULVERTAFT, J.V. (1965). A study of malignant tumours in Nigeria by short-term tissue culture. *J. Clin. Pathol.*, **18**, 261.

SAKSELA, E., TIMONEN, T., RANKI, A. & HÄYRY, P. (1979). Morphological and functional characterization of isolated effector cells responsible for human natural killer activity to fetal fibroblasts and to cultured cell line targets. *Immunol. Rev.*, **44**, 71.

SERRATE, S.A., VOSE, B.M., TIMONEN, T., ORTALDO, J.R. & HERBERMAN, R.B. (1982). Association of human natural killer cell activity against human primary tumors with large granular lymphocytes. In: *NK Cells and Other Natural Effector Cells*, p. 1055. (ed. Herberman) New York: Academic Press.

TIMONEN, T., ORTALDO, J.R. & HERBERMAN, R.B. (1981). Characteristics of human large granular lymphocytes and relationship to natural killer and K cells. *J. Exp. Med.*, **153**, 569.

TIMONEN, T., RANKI, A., SAKSELA, E. & HÄYRY, P. (1979*a*). Human natural cell-mediated cytotoxicity against fetal fibroblasts. III. Morphological and functional characterization of the effector cells. *Cell Immunol.*, **48**, 121.

TIMONEN, T., SAKSELA, E., RANKI, A. & HÄYRY, P. (1979*b*). Fractionation, morphological and functional characterization of effector cells responsible for human natural killer activity cell-line targets. *Cell Immunol.*, **48**, 133.

TIMONEN, T. & SAKSELA, E. (1980). Isolation of human NK cells by density gradient centrifugation. *J. Immunol. Methods*, **36**, 285.

UCHIDA, A. & HOSHINO, T., (1980*a*). Clinical studies on cell mediated immunity in patients with malignant disease. I. Effect of immunotherapy with OK-432 on lymphocyte subpopulation and phytomitogen responsiveness *in vitro*. *Cancer*, **45**, 476.

UCHIDA, A. & HOSHINO, T. (1980*b*). Reduction of suppressor cells in cancer patients treated with OK-432 immunotherapy. *Int. J. Cancer*, **26**, 401.

UCHIDA, A. & MICKSCHE, M. (1981*a*). Natural killer cells in carcinomatous pleural effusions. *Cancer Immunol. Immunother.*, **11**, 131.

UCHIDA, A. & MICKSCHE, M. (1981*b*). *In vitro* augmentation of natural killing activity by OK-432. *Int. J. Immunopharmacol*, **3**, 365.

UCHIDA, A. & MICKSCHE, M. (1982). Augmentation of NK cell activity in cancer patients by OK432: Activation of NK cells and reduction of suppressor cells. In: *NK Cells and Other Natural Effector Cells*, p. 1303. (ed. Herberman) New York: Academic Press.

VÀNKY, F.T., ARGOV, A.S., EINHORN, S.A. & KLEIN, E. (1980). Role of alloantigens in natural killing. Allogeneic but not autologous tumor biopsy cells are sensitive for interferon-induced cytotoxicity of human blood lymphocytes. *J. Exp. Med.*, **151**, 1151.

VERKMEISTER, J.A., PHIL, E., NIND, A.P., FLANNERY, G.R. & NAIRN, R.C. (1979). Immunoreactivity by intrinsic lymphoid cells in colorectal carcinoma. *Br. J. Cancer*, **40**, 839.

VOSE, B.M. & MOORE, M. (1980). Natural cytotoxicity in humans: Susceptibility of freshly isolated tumor cells to lysis. *J. Natl Cancer Inst.*, **65**, 257.

VOSE, B.M., VÂNKY, F., FOPP, M. & KLEIN, E. (1978). Restricted autologous lymphocytotoxicity in lung neoplasia. *Br. J. Cancer*, **38**, 375.

VOSE, B.M., VÂNKY, F. & KLEIN, E. (1977*a*). Lymphocyte cytotoxicity against autologous tumour biopsy cells in humans. *Int. J. Cancer*, **20**, 512.

VOSE, B.M., VÂNKY, F. & KLEIN, E. (1977*b*). Human tumour-lymphocyte interaction *in vitro*. V. Comparison of the reactivity of tumour-infiltrating, blood and lymph-node lymphocytes with autologous tumour cells. *Int. J. Cancer*, **20**, 895.

WAKASUGI, H., KASAHARA, T., MINATO, N., HAMURO, J., MIYATA, M., & MORIOKA, Y. (1982). *In vitro* potentiation of human natural killer cell activity by a streptococcal preparation, OK-432: Interferon and interleukin-2 participation in the stimulation with OK-432. *J. Natl Cancer Inst.*, **69**, 807.

ZARLING, J.M., ESKRA, L., BORDEN, E.C., HOROSZEWICZ, J. & CARTER, W.A. (1979). Activation of human natural killer cells cytotoxic for human leukemia cells by purified interferon. *J. Immunol.*, **123**, 63.